# Association of Glycaemic Control and Blood Pressure With Clinical Outcomes of Acute Pancreatitis in Obese Adult Men

**DOI:** 10.7759/cureus.116874

**Published:** 2026-09-24

**Authors:** Amel Isaq, Asim Fida, Sahar Hidayat Ullah, Mona Majeed, Juneda Sarfraz, Sumera Asfand, Manzoor Hussain, Shah Hussain

**Affiliations:** 1 General Medicine, Bedfordshire Hospitals NHS Foundation Trust, Bedford, GBR; 2 General Medicine, Lady Reading Hospital, Peshawar, Peshawar, PAK; 3 Emergency Department, Shifa International Hospital, Islamabad, PAK; 4 Cardiology, Punjab Institute of Cardiology, Lahore, PAK; 5 Public Health, Shaheed Zulfiqar Ali Bhutto Medical University, Islamabad, PAK; 6 Acute and General Medicine, Bedfordshire Hospitals NHS Foundation Trust, Bedford, GBR; 7 General Medicine, Mardan Medical Complex, Mardan, PAK; 8 Internal Medicine, District Headquarters Hospital Chitral, Chitral, PAK

**Keywords:** acute pancreatitis, blood pressure, clinical outcomes, disease severity, glycaemic control, hba1c, hypertension, obesity

## Abstract

Background

In obese adults, acute pancreatitis is often complicated by metabolic abnormalities, and suboptimal glycaemic control and hypertension may be linked to worse clinical outcomes and severity of disease.

Objective

To evaluate the association of glycaemic control and blood pressure status on clinical outcomes of acute pancreatitis in obese adult men.

Methodology

A prospective observational study was conducted at Pakistan Institute of Medical Sciences (PIMS), Islamabad, over one year (January 2025-December 2025). A total of 394 obese adult male patients with acute pancreatitis were enrolled using consecutive sampling. Clinical characteristics, laboratory parameters, glycaemic status, blood pressure, radiological findings, and clinical outcomes were assessed. HbA1c was categorized as <7% or ≥7% to characterize glycaemic status, while blood pressure was categorized as controlled or uncontrolled based on repeated inpatient measurements according to the prespecified threshold. Clinical outcomes were compared according to glycaemic and blood pressure status using Pearson chi-square tests. Multivariable logistic regression was performed to identify factors independently associated with severe acute pancreatitis, with adjusted odds ratios (AORs) and 95% confidence intervals (CIs) reported.

Results

Among 394 patients, 243 (61.68%) had HbA1c ≥7%, while 151 (38.32%) had HbA1c <7%. Uncontrolled blood pressure was observed in 222 (56.35%) patients compared with 172 (43.65%) with controlled blood pressure. Poor glycaemic status was associated with higher frequencies of severe acute pancreatitis (n=49; 20.16% vs. n=10; 6.62%), ICU admission (n=58; 23.87% vs. n=14; 9.27%), and in-hospital mortality (n=25; 10.29% vs. n=5; 3.31%) (all p<0.05). Uncontrolled blood pressure was associated with higher frequencies of severe acute pancreatitis (n=44; 19.82% vs. n=15; 8.72%), but the difference in in-hospital mortality was not statistically significant (n=22; 9.91% vs. n=8; 4.65%; p=0.051). In multivariable analysis, poor glycaemic control (AOR=2.84, 95% CI: 1.61-5.02, p<0.001) and uncontrolled blood pressure (AOR=2.31, 95% CI: 1.34-3.98, p=0.003) were independently associated with higher odds of severe acute pancreatitis.

Conclusion

Poor glycaemic control and uncontrolled blood pressure were associated with adverse clinical outcomes among obese adult men with acute pancreatitis. Poor glycaemic control and uncontrolled blood pressure were also independently associated with higher odds of severe acute pancreatitis in the final adjusted model. These findings support the clinical value of assessing metabolic characteristics in obese patients with acute pancreatitis, but the observational design does not establish causality or demonstrate that modifying these factors would improve outcomes. Further multicenter prospective and interventional studies are warranted to confirm these associations and determine whether targeted metabolic management can improve clinical outcomes.

## Introduction

Acute pancreatitis (AP) is among the most prevalent gastrointestinal emergencies and is defined as acute inflammation of the pancreas that ranges from a mild, self-limited illness to necrotising pancreatitis with multiple organ dysfunction and mortality [1]. The prevalence of AP has increased steadily over recent decades, a trend that has been attributed in part to the increasing prevalence of obesity, metabolic syndrome, type 2 diabetes mellitus, and hypertension worldwide [2]. Obesity is an important metabolic factor in AP because it is associated with a chronic pro-inflammatory state and may be associated with greater pancreatic inflammation and a higher frequency of local and systemic complications [3].

Hyperglycaemia is commonly observed during AP and may result from stress-induced hormonal responses, insulin resistance, and impaired pancreatic endocrine function [4]. Poor glycaemic control has been associated with greater inflammatory activity, impaired immune responses, prolonged hospitalisation, infectious complications, and mortality [5]. Hyperglycaemia may also be associated with inflammatory pathways, oxidative stress, and endothelial dysfunction, which may contribute to pancreatic injury and delayed recovery [6]. Similarly, hypertension is common among adults with obesity and metabolic abnormalities and is associated with endothelial dysfunction, microvascular injury, and impaired tissue perfusion. These metabolic and cardiovascular abnormalities may therefore represent an additional physiological burden during AP and may be associated with a more complicated clinical course [7].

Obese men may have a high burden of metabolic abnormalities, including central obesity, insulin resistance, dyslipidaemia, and hypertension [8]. The coexistence of these abnormalities and systemic inflammation may be associated with persistent organ failure, intensive care unit (ICU) admission, prolonged hospitalisation, and mortality in patients with AP [9]. Patients with obesity and metabolic abnormalities may therefore experience a more complicated course of AP, potentially reflecting the interaction between metabolic dysfunction and pancreatic inflammation [10].

The clinical severity of AP is generally assessed using the 2012 Revised Atlanta Classification, which categorises AP as mild, moderately severe, or severe according to the presence and duration of organ failure and local or systemic complications [11]. Mild AP is characterised by the absence of organ failure and local or systemic complications; moderately severe AP is characterised by transient organ failure resolving within 48 hours and/or local or systemic complications without persistent organ failure; and severe AP is defined by persistent organ failure lasting more than 48 hours [11]. Clinical and biochemical variables are also incorporated into prognostic scoring systems to identify patients at increased likelihood of adverse outcomes, including ICU admission, pancreatic necrosis, and mortality [12]. Metabolic parameters, including glycaemic control and blood pressure status, may also be associated with these outcomes and warrant further evaluation, particularly among patients with obesity [13]. Thus, clinical outcomes in AP may reflect not only the extent of pancreatic injury but also the patient's underlying metabolic and cardiovascular characteristics and their clinical status during hospitalisation [14]. Understanding the associations of potentially modifiable metabolic factors, such as glycaemic control and blood pressure, with AP outcomes may provide useful information for risk stratification and clinical management of patients at higher risk of adverse outcomes [15].

Although these interrelated metabolic factors are increasingly recognised, limited evidence has specifically examined the associations of glycaemic control and blood pressure status, both individually and in combination, with clinical outcomes of AP among obese adult men. Previous studies have frequently examined individual metabolic abnormalities separately, while the relationship between glycaemic control, blood pressure status, and AP outcomes within this specific high-risk population remains insufficiently characterised. Evaluating these associations may contribute to improved understanding of patient risk stratification and may provide clinically relevant information for management and therapeutic decision-making.

Research objective: To evaluate the associations of glycaemic control and blood pressure status with clinical outcomes of acute pancreatitis in obese adult men.

## Materials and methods

Study design

This was a prospective observational study designed to assess the association of glycaemic control and blood pressure status with clinical outcomes of acute pancreatitis (AP) among obese adult males. Participants were enrolled at hospital admission and followed prospectively throughout the index hospitalisation until discharge or in-hospital death. A standardised data-collection proforma was used to record demographic, clinical, laboratory, management, and outcome-related information. The observational design was selected to evaluate the relationship between metabolic factors and clinical outcomes without modifying or assigning any aspect of standard patient care.

Study setting

The study was conducted at Pakistan Institute of Medical Sciences (PIMS), Islamabad, a tertiary-care teaching hospital affiliated with Shaheed Zulfiqar Ali Bhutto Medical University (SZABMU), Islamabad.

Study duration

The study was conducted over one year, from January 2025 to December 2025. Patients presenting with AP during this period were screened for eligibility, consecutively enrolled, and followed during hospitalisation. Patients discharged alive from the index hospitalisation were subsequently followed for 30 days for predefined post-discharge outcomes.

Study population

The study population comprised obese adult males admitted to PIMS with a confirmed diagnosis of AP. AP was diagnosed when at least two of the following three criteria were present: (1) characteristic abdominal pain suggestive of AP; (2) serum amylase or lipase levels greater than three times the upper limit of normal; or (3) imaging findings compatible with AP on abdominal ultrasonography or contrast-enhanced computed tomography (CECT), when performed.

The severity of AP was classified according to the 2012 Revised Atlanta Classification [11]. Mild AP was defined as the absence of organ failure and local or systemic complications. Moderately severe AP was defined as transient organ failure lasting less than 48 hours and/or local or systemic complications without persistent organ failure. Severe AP was defined as persistent organ failure lasting more than 48 hours [11]. Organ failure was assessed using the Modified Marshall scoring system, as recommended by the Revised Atlanta Classification. The underlying aetiology of AP was documented, including gallstone-related, hypertriglyceridaemia-related, alcohol-related, and other identified causes.

Sample size

A total of 394 eligible obese adult males with AP were consecutively enrolled during the predefined one-year study period from January 2025 to December 2025. Because consecutive recruitment was used within a fixed study period, the final sample size was determined by the number of eligible patients presenting during that period rather than by an a priori power-based sample-size calculation. Consequently, no predefined effect size or statistical power was used to determine the recruitment target.

Among the 394 participants, 59 (14.97%) developed severe AP, which was the primary outcome used for the multivariable logistic regression analysis. To provide an event-based rationale for the number of predictors included in the regression model, the final model was restricted to five clinically relevant and prespecified predictors, corresponding to approximately 11.8 severe AP events per predictor (59/5). This restriction was applied to reduce the likelihood of model overfitting and unstable regression estimates. A two-sided significance level of 0.05 was used, and adjusted odds ratios (AORs) with 95% confidence intervals (CIs) were reported. No post-hoc selection of participants was performed according to outcome status.

Sampling technique

A consecutive non-probability sampling technique was used. All patients who fulfilled the eligibility criteria and presented during the predefined study period were consecutively enrolled. This approach minimised investigator discretion in participant selection and provided a systematic method of recruiting eligible cases from the hospital-based study population.

Eligibility criteria

Participants were eligible if they were adult males aged ≥18 years, had obesity defined as BMI ≥30 kg/m², and had a confirmed diagnosis of AP. Written informed consent was obtained before enrolment.

Patients were excluded if they had chronic pancreatitis, pancreatic malignancy, previous pancreatic surgery, AP secondary to major abdominal trauma, severe chronic liver disease, end-stage renal disease, or incomplete clinical records.

Data collection procedure

After informed consent, demographic characteristics, medical history, comorbidities, medication use, smoking history, and anthropometric measurements were recorded. Initial clinical assessment included presenting symptoms, duration of illness, vital signs, and clinical indicators relevant to disease severity.

The prespecified clinical and metabolic variables considered for descriptive assessment and potential adjustment included age, BMI, diabetes mellitus status, smoking history, dyslipidaemia, hypertension history, and AP etiology. Additional contextual variables, including dietary pattern, physical activity, medication adherence, and socioeconomic status, were documented where available to characterise potentially relevant lifestyle and behavioural factors. These variables were not automatically entered into the final regression model; inclusion in multivariable analysis was restricted to the five prespecified predictors described in the Statistical Analysis section.

Blood pressure was assessed using standard hospital procedures. Glycaemic status was assessed using admission blood glucose and HbA1c. Laboratory investigations included complete blood count, serum amylase, serum lipase, liver function tests, renal function tests, serum electrolytes, lipid profile, and C-reactive protein. Abdominal ultrasonography and CECT were reviewed when clinically indicated to assess pancreatic inflammation, necrosis, and local complications.

Patients received treatment according to institutional standards, and their clinical course was prospectively recorded throughout hospitalisation. Patients who survived the index hospitalisation were followed for 30 days after discharge through outpatient visits or structured telephone follow-up. Patients who died during the index hospitalisation were not included in post-discharge follow-up because they were not eligible for follow-up after discharge.

The 30-day post-discharge outcomes included readmission, recurrence of AP, delayed pancreatic complications, requirement for further endoscopic or surgical intervention, and post-discharge mortality. Adverse events and additional interventions were also documented.

Assessment of glycaemic control

HbA1c measured at admission was used as an indicator of pre-existing glycaemic status rather than as a measure of the acute metabolic response to pancreatitis. Participants were categorised into two groups according to an HbA1c threshold of 7.0%: HbA1c <7.0% and HbA1c ≥7.0%.

For the purpose of this study, HbA1c <7.0% was categorised as adequate glycaemic control, whereas HbA1c ≥7.0% was categorised as poor glycaemic control. This threshold was applied to the entire study cohort, irrespective of previously diagnosed diabetes mellitus, because the analysis aimed to examine the association between the degree of chronic glycaemic exposure and AP outcomes rather than to define diabetes mellitus itself. Thus, HbA1c ≥7.0% was used as an exposure classification and was not considered synonymous with a clinical diagnosis of diabetes mellitus. Diabetes mellitus status was recorded separately based on the participant's established medical history/clinical documentation.

Assessment of blood pressure

Blood pressure was measured during hospitalisation using a calibrated Medico BPM-35 automated sphygmomanometer with an appropriately sized cuff, following standard clinical measurement procedures. Patients were allowed to rest before measurement and were assessed in a comfortable seated position with the back supported, feet appropriately positioned, and the arm supported at heart level. Systolic blood pressure (SBP) and diastolic blood pressure (DBP) were recorded in millimetres of mercury (mmHg). Blood pressure was assessed at admission and subsequently during routine inpatient clinical assessments as part of standard hospital care. To minimise misclassification based on a single transient admission reading, the blood pressure exposure used for analysis was determined from repeated measurements obtained during the index hospitalisation. Participants were classified as having uncontrolled blood pressure when repeated inpatient measurements demonstrated SBP ≥140 mmHg and/or DBP ≥90 mmHg, whereas participants whose recorded measurements remained below these thresholds were classified as having controlled blood pressure. The same prespecified classification criteria were applied consistently to all participants before outcome analysis. Antihypertensive medications administered during hospitalisation were recorded as part of routine clinical management and were not assigned or modified by the study investigators.

Outcome assessment

Clinical outcomes were assessed prospectively during the index hospitalisation. The primary outcome for the multivariable analysis was severe AP, defined according to the Revised Atlanta Classification as persistent organ failure lasting more than 48 hours. Other clinically important outcomes included persistent organ failure, ICU admission, and in-hospital mortality.

Secondary outcomes included length of hospital stay, pancreatic necrosis, and requirement for invasive management, including endoscopic, radiological, or surgical intervention where clinically indicated. The predefined outcomes were used to evaluate the association of glycaemic control and blood pressure status with the clinical course of AP.

Data management and quality control

All data were entered into a structured data-collection form and checked for completeness, consistency, and accuracy before statistical analysis. Each participant was assigned a unique study identification code to maintain confidentiality. Regular verification and consistency checks were performed to minimise data-entry errors and improve data quality.

Statistical analysis

Statistical analysis was performed using IBM SPSS Statistics for Windows, Version 27.0 (IBM Corp., released 2020; Armonk, NY, USA). Continuous variables were summarised as mean ± standard deviation (SD), whereas categorical variables were presented as frequencies and percentages. The Pearson chi-square test was used to compare categorical variables between groups.

For multivariable logistic regression, severe AP was specified as the binary dependent outcome (yes/no). Given the 59 severe AP events, the number of predictors was deliberately restricted to reduce the possibility of model overfitting and unstable estimates. Candidate variables were considered a priori on the basis of the primary study objective, established clinical relevance, and their potential role as confounding factors. However, not all recorded or initially considered variables were entered into the final regression model.

The final multivariable model included five prespecified predictors: poor glycaemic control (HbA1c ≥7%), uncontrolled blood pressure, BMI ≥40 kg/m², diabetes mellitus, and hypertriglyceridaemia-related AP. The corresponding reference categories were adequate glycaemic control (HbA1c <7%), controlled blood pressure, BMI <40 kg/m², absence of diabetes mellitus, and AP not attributed to hypertriglyceridaemia.

The selection of these five variables was specified before the final regression analysis and was based on their direct relevance to the study objective and potential clinical confounding. Variables such as age, smoking history, dyslipidaemia, hypertension history, dietary pattern, physical activity, medication adherence, and socioeconomic status were recorded or considered clinically relevant but were not included in the final model because the number of severe AP events was limited and inclusion of a larger number of predictors could increase model instability.

Missing data were assessed before analysis. There were no missing observations for the variables included in the final multivariable model; therefore, no missing-data imputation was required. The logistic regression model was evaluated for the appropriateness of the included predictors and the stability of the estimated associations. Adjusted odds ratios (AORs) with 95% confidence intervals (CIs) were reported. A two-tailed p-value <0.05 was considered statistically significant.

Ethical considerations

Ethical approval was obtained from the Ethical Review Committee of Shaheed Zulfiqar Ali Bhutto Medical University (SZABMU), Islamabad (F.1-1/2024/ERB/SZABMU/1872), before commencement of the study. The study was conducted at PIMS, the affiliated tertiary-care teaching hospital of SZABMU. Written informed consent was obtained from all participants before enrolment. Participant privacy and confidentiality were maintained throughout the study, and the research was conducted in accordance with the ethical principles of the Declaration of Helsinki.

## Results

Baseline characteristics of 394 obese adult men with acute pancreatitis showed a mean age of 52.6 ± 11.4 years, with the majority aged 41-60 years (n=214; 54.31%), as shown in Table 1. Most patients had Class I obesity (n=176; 44.67%), while diabetes mellitus and hypertension were present in 238 (60.41%) and 251 (63.71%) patients, respectively. Poor dietary pattern was observed in 218 (55.33%), low physical activity in 247 (62.69%), and poor medication adherence in 96 (40.34%) patients. Low socioeconomic status was reported in 171 (43.40%) participants. The most common symptoms were abdominal pain (n=394; 100%), nausea (n=312; 79.19%), and vomiting (n=276; 70.05%). According to the 2012 Revised Atlanta Classification [11], 214 (54.31%) patients had mild acute pancreatitis, 121 (30.71%) had moderately severe acute pancreatitis, and 59 (14.97%) had severe acute pancreatitis. Because severe acute pancreatitis was defined by persistent organ failure lasting more than 48 hours, 59 patients also met the criterion for persistent organ failure.

**Table 1:** Baseline demographic, clinical, biochemical, and disease characteristics of obese adult male patients with acute pancreatitis (n = 394). Note: Continuous variables are presented as mean ± SD, and categorical variables as frequency (percentage). Percentages are based on the total study population (n = 394), except for variables assessed among specific subgroups. Poor medication adherence was calculated among patients receiving chronic therapy (n = 238).

Variable	n (%) / Mean ± SD
Demographic characteristics	
Age (years)	52.6 ± 11.4
18-40 years	92 (23.35%)
41-60 years	214 (54.31%)
>60 years	88 (22.34%)
Anthropometric characteristics	
Body mass index (kg/m²)	34.7 ± 3.9
Obesity Class I (30-34.9 kg/m²)	176 (44.67%)
Obesity Class II (35-39.9 kg/m²)	142 (36.04%)
Obesity Class III (≥40 kg/m²)	76 (19.29%)
Comorbidities and medical history	
Diabetes mellitus	238 (60.41%)
Duration of diabetes (years)	7.4 ± 3.6
Hypertension	251 (63.71%)
Dyslipidaemia	198 (50.25%)
Smoking history	167 (42.39%)
Previous pancreatitis	74 (18.78%)
Lifestyle and behavioural characteristics	
Poor dietary pattern (high-fat/high-calorie diet)	218 (55.33%)
Low physical activity level	247 (62.69%)
Poor medication adherence among patients receiving chronic therapy	96 (40.34%)
Socioeconomic characteristics	
Low socioeconomic status	171 (43.40%)
Middle/high socioeconomic status	223 (56.60%)
Presenting symptoms	
Duration of symptoms before admission (days)	3.8 ± 1.7
Abdominal pain	394 (100.00%)
Nausea	312 (79.19%)
Vomiting	276 (70.05%)
Fever	128 (32.49%)
Abdominal tenderness	349 (88.58%)
Vital signs at admission	
Heart rate (beats/min)	101.4 ± 16.2
Respiratory rate (breaths/min)	22.1 ± 4.5
Body temperature (°C)	37.9 ± 0.8
Systolic blood pressure (mmHg)	148.3 ± 18.6
Diastolic blood pressure (mmHg)	91.7 ± 12.4
Baseline laboratory parameters	
HbA1c (%)	8.1 ± 1.9
Random blood glucose (mg/dL)	212.6 ± 68.4
Hemoglobin (g/dL)	13.1 ± 1.8
White blood cell count (×10⁹/L)	14.6 ± 4.5
Platelet count (×10⁹/L)	256.8 ± 74.2
Serum amylase (U/L)	726.4 ± 281.7
Serum lipase (U/L)	914.8 ± 365.3
ALT (U/L)	76.9 ± 34.8
AST (U/L)	69.4 ± 31.6
Total bilirubin (mg/dL)	1.6 ± 0.8
Alkaline phosphatase (U/L)	142.5 ± 48.6
Serum creatinine (mg/dL)	1.21 ± 0.39
Blood urea nitrogen (mg/dL)	24.8 ± 9.7
Sodium (mmol/L)	138.2 ± 4.6
Potassium (mmol/L)	4.1 ± 0.5
Total cholesterol (mg/dL)	218.4 ± 42.6
LDL cholesterol (mg/dL)	137.8 ± 35.4
HDL cholesterol (mg/dL)	41.6 ± 9.2
Triglycerides (mg/dL)	276.5 ± 118.7
C-reactive protein (mg/L)	78.2 ± 32.6
Etiology of acute pancreatitis	
Gallstone pancreatitis	168 (42.64%)
Hypertriglyceridaemia	102 (25.89%)
Alcohol-related pancreatitis	74 (18.78%)
Other/idiopathic	50 (12.69%)
Radiological findings	
Pancreatic edema	263 (66.75%)
Peripancreatic fluid collection	119 (30.20%)
Pancreatic necrosis	68 (17.26%)
Pancreatic pseudocyst	24 (6.09%)
Disease severity (Revised Atlanta Classification)	
Mild acute pancreatitis	214 (54.31%)
Moderately severe acute pancreatitis	121 (30.71%)
Severe acute pancreatitis	59 (14.97%)

Radiological evaluation showed pancreatic oedema in 263 (66.75%) patients, peripancreatic fluid collection in 119 (30.20%), pancreatic necrosis in 68 (17.26%), and pancreatic pseudocyst in 24 (6.09%). A representative contrast-enhanced computed tomography (CECT) image demonstrating pancreatic inflammatory changes and peripancreatic involvement is presented in Figure 1.

**Figure 1:**
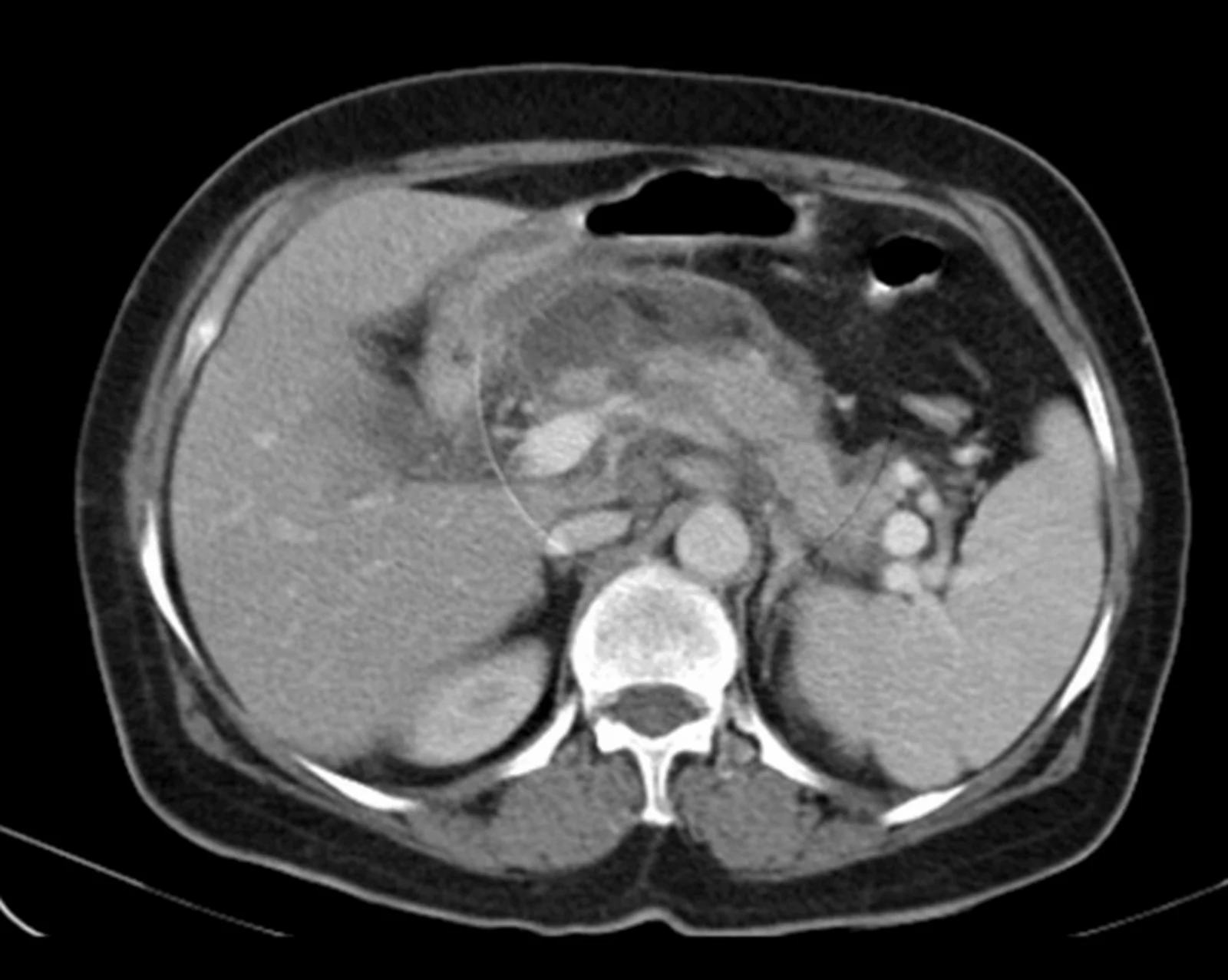
Contrast-enhanced computed tomography (CECT) demonstrating acute pancreatitis with pancreatic enlargement, heterogeneous enhancement, and prominent peripancreatic inflammatory changes.

Among 394 patients, 243 (61.68%) had poor glycaemic control (HbA1c ≥7%), whereas 151 (38.32%) had adequate glycaemic control (Table 2). Uncontrolled blood pressure was present in 222 (56.35%) patients, while 172 (43.65%) had controlled blood pressure.

**Table 2:** Glycaemic control and blood pressure classification (n=394)

Variables		n (%)
Glycaemic control	HbA1c <7% (adequate control)	151 (38.32%)
	HbA1c ≥7% (poor control)	243 (61.68%)
Blood pressure status	Controlled BP	172 (43.65%)
	Uncontrolled BP	222 (56.35%)

Patients with poor glycaemic control had higher frequencies of severe acute pancreatitis (20.16% vs. 6.62%), persistent organ failure (20.16% vs. 6.62%), ICU admission (23.87% vs. 9.27%), pancreatic necrosis (21.81% vs. 9.93%), hospital stay >7 days (39.09% vs. 27.81%), invasive intervention (22.22% vs. 11.92%), and in-hospital mortality (10.29% vs. 3.31%) compared with patients with adequate glycaemic control. The differences were statistically significant for all evaluated outcomes (p≤0.022) (Table 3).

**Table 3:** Association of glycaemic control with clinical outcomes (Pearson chi-square test).

Variables	HbA1c <7% (n=151)	HbA1c ≥7% (n=243)	χ²	p-value
Severe acute pancreatitis	10 (6.62%)	49 (20.16%)	13.41	<0.001
Persistent organ failure	10 (6.62%)	49 (20.16%)	13.41	<0.001
ICU admission	14 (9.27%)	58 (23.87%)	13.29	<0.001
Pancreatic necrosis	15 (9.93%)	53 (21.81%)	9.20	0.002
Hospital stay >7 days	42 (27.81%)	95 (39.09%)	5.22	0.022
Invasive intervention required	18 (11.92%)	54 (22.22%)	6.62	0.010
In-hospital mortality	5 (3.31%)	25 (10.29%)	6.44	0.011

Patients with uncontrolled blood pressure had higher frequencies of severe acute pancreatitis (19.82% vs. 8.72%), persistent organ failure (19.82% vs. 8.72%), pancreatic necrosis (22.07% vs. 11.05%), ICU admission (22.52% vs. 12.79%), and hospital stay >7 days (40.99% vs. 26.74%) compared with patients with controlled blood pressure. These differences were statistically significant (p≤0.013). Differences in invasive intervention (21.62% vs. 13.95%; p=0.051) and in-hospital mortality (9.91% vs. 4.65%; p=0.051) did not reach statistical significance (Table 4).

**Table 4:** Association of blood pressure status with clinical outcomes (Pearson chi-square test).

Variables	Controlled BP (n=172)	Uncontrolled BP (n=222)	χ²	p-value
Severe acute pancreatitis	15 (8.72%)	44 (19.82%)	9.38	0.002
Persistent organ failure	15 (8.72%)	44 (19.82%)	9.38	0.002
Pancreatic necrosis	19 (11.05%)	49 (22.07%)	8.25	0.004
ICU admission	22 (12.79%)	50 (22.52%)	6.15	0.013
Hospital stay >7 days	46 (26.74%)	91 (40.99%)	8.67	0.003
Invasive intervention required	24 (13.95%)	48 (21.62%)	3.82	0.051
In-hospital mortality	8 (4.65%)	22 (9.91%)	3.81	0.051

The poor glycaemic control plus uncontrolled blood pressure group had the highest frequencies of severe acute pancreatitis (n=40; 23.95%), ICU admission (n=44; 26.35%), and in-hospital mortality (n=20; 11.98%), compared with the good glycaemic control plus controlled blood pressure group (6.25%, 8.33%, and 3.13%, respectively). The differences across the four groups were statistically significant for severe acute pancreatitis (χ²=19.46, p<0.001), ICU admission (χ²=15.64, p=0.001), and in-hospital mortality (χ²=8.62, p=0.035) (Table 5).

**Table 5:** Association of combined glycaemic control and blood pressure status with major clinical outcomes (Pearson chi-square test).

Variables	Good GC + Controlled BP (n=96)	Poor GC + Controlled BP (n=76)	Good GC + Uncontrolled BP (n=55)	Poor GC + Uncontrolled BP (n=167)	χ²	p-value
Severe acute pancreatitis	6 (6.25%)	9 (11.84%)	4 (7.27%)	40 (23.95%)	19.46	<0.001
ICU admission	8 (8.33%)	14 (18.42%)	6 (10.91%)	44 (26.35%)	15.64	0.001
In-hospital mortality	3 (3.13%)	5 (6.58%)	2 (3.64%)	20 (11.98%)	8.62	0.035

Of the 394 patients enrolled in the study, 30 died during the index hospitalisation, while 364 patients survived to discharge and were therefore eligible for the planned 30-day post-discharge follow-up. The 30 in-hospital deaths were excluded from the post-discharge follow-up analysis because they were not alive at the time of discharge and consequently could not contribute to outcomes occurring after discharge. The 364 survivors were distributed across the four glycaemic control and blood pressure subgroups as follows: 93 with good glycaemic control and controlled blood pressure, 71 with poor glycaemic control and controlled blood pressure, 53 with good glycaemic control and uncontrolled blood pressure, and 147 with poor glycaemic control and uncontrolled blood pressure. During 30-day follow-up, the poor glycaemic control with uncontrolled blood pressure group had the highest rates of readmission (n=17; 11.56%), recurrent acute pancreatitis (n=13; 8.84%), delayed pancreatic complications (n=10; 6.80%), additional endoscopic intervention (n=7; 4.76%), surgical intervention (n=4; 2.72%), and post-discharge mortality (n=2; 1.36%), shown in Table 6. However, none of these differences was statistically significant (all p>0.05).

**Table 6:** Thirty-day post-discharge follow-up outcomes among the 364 patients who survived to discharge, according to glycaemic control and blood pressure status The original cohort consisted of 394 patients. Thirty patients died during the index hospitalization and were excluded from post-discharge follow-up analysis. Therefore, Table 6 includes only the 364 patients who survived to discharge and were eligible for 30-day follow-up. The subgroup denominators sum to 364 (93 + 71 + 53 + 147 = 364). Percentages are calculated using the corresponding subgroup denominator.

Variables	Good GC + Controlled BP (n=93)	Poor GC + Controlled BP (n=71)	Good GC + Uncontrolled BP (n=53)	Poor GC + Uncontrolled BP (n=147)	χ²	p-value
Readmission	5 (5.38%)	7 (9.86%)	5 (9.43%)	17 (11.56%)	2.61	0.456
Recurrent acute pancreatitis	3 (3.23%)	6 (8.45%)	4 (7.55%)	13 (8.84%)	2.99	0.393
Delayed pancreatic complications	3 (3.23%)	5 (7.04%)	4 (7.55%)	10 (6.80%)	1.79	0.618
Additional endoscopic intervention	2 (2.15%)	3 (4.23%)	2 (3.77%)	7 (4.76%)	1.08	0.781
Additional surgical intervention	1 (1.08%)	2 (2.82%)	1 (1.89%)	4 (2.72%)	0.88	0.830
Post-discharge mortality within 30 days among discharged patients	1 (1.08%)	2 (2.82%)	1 (1.89%)	2 (1.36%)	0.88	0.830

Multivariable logistic regression analysis showed that poor glycaemic control was independently associated with higher odds of severe acute pancreatitis (AOR=2.84, 95% CI: 1.61-5.02, p<0.001). Uncontrolled blood pressure was also independently associated with severe acute pancreatitis (AOR=2.31, 95% CI: 1.34-3.98, p=0.003). Similarly, BMI ≥40 kg/m² (AOR=1.92, 95% CI: 1.08-3.41, p=0.026), diabetes mellitus (AOR=1.74, 95% CI: 1.03-2.95, p=0.039), and hypertriglyceridaemia-related pancreatitis (AOR=2.16, 95% CI: 1.23-3.81, p=0.007) were independently associated with severe acute pancreatitis after adjustment for the other variables included in the model (Table 7).

**Table 7:** Multivariable logistic regression analysis of predictors of severe acute pancreatitis, with adjusted odds ratios and 95% confidence intervals

Variables	Adjusted OR	95% CI	Wald χ²	p-value
Poor glycaemic control (HbA1c ≥7%)	2.84	1.61-5.02	13.48	<0.001
Uncontrolled blood pressure	2.31	1.34-3.98	8.93	0.003
BMI ≥40 kg/m²	1.92	1.08-3.41	4.95	0.026
Diabetes mellitus	1.74	1.03-2.95	4.27	0.039
Hypertriglyceridaemia-related pancreatitis	2.16	1.23-3.81	7.28	0.007

## Discussion

The aim of this prospective observational study was to assess the association of glycaemic control and blood pressure status with clinical outcomes of acute pancreatitis (AP) in obese adult male patients. A total of 394 adult male patients with AP were included, with a mean age of 52.6 ± 11.4 years and a mean BMI of 34.7 ± 3.9 kg/m². The majority of patients were aged 41-60 years (n=214; 54.31%), and Class I obesity was the most frequent obesity category (n=176; 44.67%). Diabetes mellitus and hypertension were present in 238 (60.41%) and 251 (63.71%) patients, respectively. Gallstone pancreatitis was the most common aetiology (n=168; 42.64%), while severe AP according to the Revised Atlanta Classification was observed in 59 (14.97%) patients. Overall, these findings demonstrate a substantial metabolic burden among obese patients presenting with AP. Previous evidence has shown that obesity is associated with more severe pancreatic disease and systemic complications, potentially through mechanisms involving visceral adiposity, inflammation, and metabolic dysfunction. Shin et al. reported a significant association between obesity and increased severity and complications of AP, supporting the clinical relevance of metabolic assessment in this population [16]. However, because the present study was observational, these findings should be interpreted as associations rather than evidence that obesity or other metabolic abnormalities directly cause adverse AP outcomes.

This study showed that poor glycaemic control was common, with 243 (61.68%) patients having HbA1c ≥7% and 151 (38.32%) having HbA1c <7%. Patients with HbA1c ≥7% had significantly higher frequencies of severe AP (n=49; 20.16% vs. n=10; 6.62%), persistent organ failure (n=49; 20.16% vs. n=10; 6.62%), ICU admission (n=58; 23.87% vs. n=14; 9.27%), pancreatic necrosis (n=53; 21.81% vs. n=15; 9.93%), hospital stay >7 days (n=95; 39.09% vs. n=42; 27.81%), invasive intervention (n=54; 22.22% vs. n=18; 11.92%), and in-hospital mortality (n=25; 10.29% vs. n=5; 3.31%). These findings indicate an association between poorer chronic glycaemic status and several adverse clinical outcomes in this cohort, but they do not establish that poor glycaemic control caused progression of AP. Han et al. similarly reported that elevated HbA1c was associated with AP severity and clinical outcomes, suggesting that chronic glycaemic status may have prognostic relevance [17]. In addition, a previous meta-analysis found that pre-diabetes was associated with systemic complications and higher ICU admission among patients with AP, which is consistent with the direction of the present findings [18].

The HbA1c threshold of ≥7% was applied to the entire cohort to characterise chronic glycaemic status rather than to define diabetes mellitus. HbA1c reflects longer-term glycaemic exposure and is therefore less affected by the acute physiological stress of AP than a single admission glucose measurement. Diabetes mellitus was assessed and recorded separately as a clinical comorbidity. Accordingly, the HbA1c classification should not be interpreted as indicating that every participant with HbA1c ≥7% had established diabetes. Nevertheless, applying this threshold to participants without previously diagnosed diabetes may identify individuals with previously unrecognised dysglycaemia and may introduce some degree of classification uncertainty. This limitation has therefore been acknowledged in the revised manuscript.

In the present study, uncontrolled blood pressure was also associated with poorer clinical outcomes. Severe AP occurred in 44 (19.82%) patients with uncontrolled blood pressure compared with 15 (8.72%) patients with controlled blood pressure. Similarly, uncontrolled blood pressure was associated with higher frequencies of persistent organ failure (n=44; 19.82% vs. n=15; 8.72%), pancreatic necrosis (n=49; 22.07% vs. n=19; 11.05%), ICU admission (n=50; 22.52% vs. n=22; 12.79%), hospital stay >7 days (n=91; 40.99% vs. n=46; 26.74%), invasive intervention (n=48; 21.62% vs. n=24; 13.95%), and mortality (n=22; 9.91% vs. n=8; 4.65%). These results indicate an association between higher inpatient blood pressure measurements and adverse AP outcomes, but the interpretation requires particular caution because blood pressure was assessed during acute hospitalisation. Acute pain, systemic inflammation, stress responses, fluid shifts, and other features of AP may transiently elevate blood pressure. Therefore, the observed relationship may partly reflect acute illness severity or reverse temporal relationships rather than the effect of pre-existing hypertension itself. In addition, classification based on repeated in-hospital measurements may result in some degree of exposure misclassification. Recent evidence suggests that pre-existing hypertension may be associated with poorer outcomes in AP. A systematic review and meta-analysis reported a significant association between hypertension and mortality, although the association with severe AP was not statistically significant [13]. Thus, the present findings should be considered hypothesis-generating with respect to the relationship between blood pressure status and AP severity, and confirmation using pre-admission or longitudinal blood pressure measurements would strengthen future studies.

The analysis of combined glycaemic and blood pressure status further demonstrated that the group with poor glycaemic control and uncontrolled blood pressure had the highest observed frequencies of severe AP (n=40; 23.95%), ICU admission (n=44; 26.35%), and in-hospital mortality (n=20; 11.98%), compared with 6 (6.25%), 8 (8.33%), and 3 (3.13%), respectively, among patients with good glycaemic control and controlled blood pressure. These findings suggest that the coexistence of adverse metabolic characteristics was associated with a greater burden of adverse outcomes. However, the four-group analysis should not be interpreted as demonstrating an additive or synergistic causal effect, because the observational design does not permit determination of causality or interaction. Evidence concerning individual components of metabolic syndrome has similarly suggested associations with poorer outcomes in AP, supporting the value of comprehensive metabolic assessment rather than consideration of isolated metabolic characteristics [18].

The independent associations of metabolic factors with severe AP were further examined using multivariable logistic regression. Poor glycaemic control was associated with higher odds of severe disease (AOR=2.84, 95% CI: 1.61-5.02, p<0.001), followed by uncontrolled blood pressure (AOR=2.31, 95% CI: 1.34-3.98, p=0.003). BMI ≥40 kg/m² (AOR=1.92, 95% CI: 1.08-3.41, p=0.026), diabetes mellitus (AOR=1.74, 95% CI: 1.03-2.95, p=0.039), and hypertriglyceridaemia-related pancreatitis (AOR=2.16, 95% CI: 1.23-3.81, p=0.007) were also independently associated with severe AP. These findings indicate that the included metabolic and clinical characteristics retained independent statistical associations with severe AP after adjustment for the other variables included in the final model; they should not be interpreted as independent causal risk factors. A systematic review and meta-analysis has reported that pre-existing diabetes is associated with increased complications, ICU admission, renal failure, and longer hospitalisation among patients with AP [18]. The present findings therefore support the potential clinical value of identifying patients with adverse glycaemic and blood pressure profiles, while prospective interventional studies are required to determine whether modifying these factors changes AP outcomes.

The apparent discrepancy between severe AP and persistent organ failure counts identified during revision was also addressed. According to the Revised Atlanta Classification, severe AP is defined by persistent organ failure lasting >48 hours. Accordingly, the persistent organ failure counts were corrected to correspond with the 59 severe AP cases, with 10 versus 49 cases according to HbA1c status and 15 versus 44 cases according to blood pressure status. The relevant percentages, chi-square statistics, and p-values were recalculated from the corrected event counts. Several previously reported chi-square values and p-values were also corrected after verification against the displayed frequencies. These revisions improve the internal consistency between the Methods, Results, tables, and Discussion without changing the underlying study design or the overall direction of the findings.

The regression analysis should also be interpreted in the context of variable selection. Although a broader range of demographic, clinical, lifestyle, biochemical, and treatment-related variables was recorded during data collection, the final multivariable model was restricted to the five prespecified variables based on the available number of severe AP events. Therefore, the variables described as recorded or considered in the Methods should not be interpreted as having all been entered simultaneously into the final regression model. This distinction has been clarified to avoid overstating the extent of statistical adjustment. Consequently, residual confounding from variables not included in the final model cannot be excluded.

Strengths and limitations

This study has several strengths. First, its prospective observational design allowed clinical, metabolic, biochemical, radiological, and outcome information to be collected systematically during the study period rather than relying entirely on retrospective records. Second, the study included a relatively large cohort of 394 obese adult men with a confirmed diagnosis of AP, providing sufficient observations for detailed assessment of clinically relevant outcomes. Third, AP diagnosis and severity were determined using established diagnostic and classification criteria, including the Revised Atlanta Classification and assessment of organ failure using the Modified Marshall system. Fourth, the study evaluated several clinically relevant metabolic characteristics, including HbA1c, blood pressure status, BMI, diabetes mellitus, and hypertriglyceridaemia, together with relevant clinical, biochemical, radiological, and lifestyle-related variables. Fifth, the use of multivariable logistic regression permitted estimation of adjusted associations between prespecified metabolic characteristics and severe AP while accounting for the other variables included in the final model. Finally, 30-day post-discharge follow-up of survivors provided additional information regarding readmission, recurrent AP, delayed complications, interventions, and post-discharge mortality.

Several limitations should nevertheless be considered when interpreting the findings. The study was conducted at a single tertiary-care hospital and included only obese adult men; therefore, the findings may not be directly generalisable to women, non-obese patients, younger populations, community settings, or other healthcare systems. The use of consecutive non-probability sampling may also have introduced selection bias. The observational design precludes determination of causality, and the reported adjusted odds ratios represent associations rather than causal effects.

An important limitation concerns blood pressure assessment. Blood pressure was classified using repeated measurements obtained during hospitalisation. Although repeated measurements are preferable to relying on a single reading, blood pressure during an acute episode of pancreatitis may be influenced by pain, systemic inflammatory responses, stress, fluid status, medications, and the severity of acute illness itself. Consequently, the inpatient blood pressure category may not accurately represent the patient's usual pre-morbid or long-term blood pressure status. This creates the possibility of exposure misclassification and reverse temporal interpretation, whereby more severe illness may contribute to abnormal blood pressure rather than abnormal pre-existing blood pressure contributing to severe illness. Future studies should incorporate pre-admission blood pressure records, ambulatory blood pressure measurements, or longitudinal measurements obtained after recovery to better characterise chronic blood pressure status.

Another methodological consideration is the use of HbA1c ≥7% to classify poor glycaemic control in the entire cohort, including participants without previously diagnosed diabetes. The threshold was used to characterise chronic glycaemic exposure and was not intended to diagnose diabetes; diabetes status was recorded separately. Nevertheless, this approach may identify previously unrecognised dysglycaemia among participants without known diabetes and may therefore introduce some degree of classification uncertainty. HbA1c is also not completely independent of chronic comorbidities and other physiological factors. Future studies could strengthen this assessment by combining HbA1c with documented pre-admission glycaemic measurements, prior diabetes records, or standardised diagnostic testing where clinically appropriate.

The study also has limitations related to confounding and model specification. A range of demographic, lifestyle, clinical, and treatment-related variables was recorded, but the final multivariable logistic regression model included only five prespecified variables because the number of severe AP events limited the number of variables that could reasonably be entered into the model. Thus, recording a variable did not imply that it was included in the final regression model. Residual confounding from variables not included in the final model, including detailed dietary patterns, physical activity, socioeconomic circumstances, medication adherence, duration and treatment of chronic metabolic disease, and other unmeasured clinical characteristics, cannot be excluded. In addition, diabetes mellitus and HbA1c-based glycaemic classification may be correlated, which makes assessment of multicollinearity important when interpreting the regression estimates. Accordingly, the regression findings should be regarded as adjusted associations within the specified model rather than definitive independent causal effects.

During manuscript revision, an inconsistency between the reported number of severe AP cases and persistent organ failure cases was identified and corrected. Because persistent organ failure lasting >48 hours defines severe AP according to the Revised Atlanta Classification, the persistent organ failure counts were aligned with the 59 severe AP cases. The corresponding percentages and statistical comparisons were recalculated. In addition, several chi-square statistics and p-values were rechecked against the displayed event counts and corrected where discrepancies were identified. These corrections improve the numerical and conceptual consistency of the Results and tables.

The study was also limited by the absence of an a priori power calculation because all eligible cases presenting during the fixed one-year recruitment period were consecutively enrolled. Although the final sample included 394 participants and 59 severe AP events, the number of events restricted the complexity of the multivariable model. The final analysis was therefore deliberately limited to five prespecified predictors rather than selecting a larger number of variables solely on the basis of statistical significance. This approach reduced the risk of excessive model complexity, but it also means that potentially relevant associations involving variables not included in the final model could not be fully evaluated.

Finally, although 30-day post-discharge follow-up provided useful information regarding delayed outcomes, the follow-up period was relatively short and therefore cannot address longer-term recurrence, persistent pancreatic complications, or sustained metabolic outcomes. Larger multicenter prospective studies involving both sexes, broader BMI categories, standardised pre-morbid blood pressure assessment, more detailed longitudinal glycaemic measurements, and longer follow-up are warranted to validate these findings and clarify the temporal relationships between metabolic characteristics and AP outcomes.

## Conclusions

Poor glycaemic control and uncontrolled blood pressure were significantly associated with adverse clinical outcomes among obese adult men with acute pancreatitis. Severe acute pancreatitis, persistent organ failure, ICU admission, pancreatic necrosis, prolonged hospitalisation, and in-hospital mortality occurred more frequently among patients with poor glycaemic control and those with uncontrolled blood pressure. In multivariable analysis, poor glycaemic control, uncontrolled blood pressure, BMI ≥40 kg/m², diabetes mellitus, and hypertriglyceridaemia-related pancreatitis were independently associated with higher odds of severe acute pancreatitis within the final adjusted model.

These findings support the clinical value of early assessment of metabolic characteristics in obese patients with acute pancreatitis. However, because of the observational design, these associations should not be interpreted as causal, and the study cannot determine whether modifying glycaemic control or blood pressure would improve clinical outcomes. The findings should therefore be validated in larger multicenter prospective studies incorporating standardised pre-morbid blood pressure assessment, longitudinal glycaemic measurements, and longer follow-up, with interventional studies needed to determine whether targeted metabolic management improves outcomes.
